# *PIK3CA* amplification is associated with poor prognosis among patients with curatively resected esophageal squamous cell carcinoma

**DOI:** 10.18632/oncotarget.8749

**Published:** 2016-04-15

**Authors:** Hyo Song Kim, Seung Eun Lee, Yoon Sung Bae, Dae Joon Kim, Chang Geol Lee, Jin Hur, Hyunsoo Chung, Jun Chul Park, Sung Kwan Shin, Sang Kil Lee, Yong Chan Lee, Hye Ryun Kim, Young Mog Shim, Susan S. Jewell, Hyunki Kim, Yoon-La Choi, Byoung Chul Cho

**Affiliations:** ^1^ Division of Medical Oncology, Department of Internal Medicine, Yonsei University College of Medicine, Seoul, Korea; ^2^ Department of Pathology, Konkuk University School of Medicine, Konkuk University Medical Center, Seoul, Korea; ^3^ Department of Pathology, Yonsei University College of Medicine, Seoul, Korea; ^4^ Department of Thoracic and Cardiovascular Surgery, Yonsei University College of Medicine, Seoul, Korea; ^5^ Department of Radiation Oncology, Yonsei University College of Medicine, Seoul, Korea; ^6^ Department of Radiology, Yonsei University College of Medicine, Seoul, Korea; ^7^ Division of Gastroenterology, Department of Internal Medicine, Yonsei University College of Medicine, Seoul, Korea; ^8^ Department of Thoracic Surgery, Samsung Medical Center, Sungkyunkwan University School of Medicine, Seoul, Korea; ^9^ Abbott Molecular Laboratories, Des Plaines, IL, United States; ^10^ Department of Pathology, Samsung Medical Center, Sungkyunkwan University School of Medicine, Seoul, Korea

**Keywords:** PIK3CA, esophageal squamous cell carcinoma, amplification, mutation, fluorescent in situ hybridization

## Abstract

To investigate the clinicopathologic characteristics and the prognostic impact of *PIK3CA* gene amplification in curatively resected esophageal squamous cell carcinoma (ESCC). Using 534 curatively resected ESCCs, the *PIK3CA* gene copy number was evaluated with fluorescent in situ hybridization. *PIK3CA* amplification was defined as *PIK3CA*/centromere 3 ratio is ≥ 2.0 or average number of *PIK3CA* signals/tumor cell nucleus ≥ 5.0. *PIK3CA* mutations in exon 9 and 20, encoding the highly conserved helical and kinase domains were assessed by direct sequencing in 388 cases. *PIK3CA* amplification was detected in 56 (10.5%) cases. *PIK3CA* amplification was significantly associated with higher T-stage (P=0.026) and pathologic stage (P=0.053). *PIK3CA* amplification showed a significantly shorter disease free survival (DFS) compared with that of non-amplified group (33.4 vs 63.1 months, P=0.019). After adjusting for gender, tumor location, pathologic stage, histologic grade and adjuvant treatment, *PIK3CA* amplification was significantly associated with a shorter DFS (adjusted hazard ratio [AHR] 1.53; 95% CI, 1.10-2.17; P=0.02). Though the statistical insignificance, *PIK3CA* amplification showed tendency of shorter OS (52.1 vs 96.5 moths, P=0.116). *PIK3CA* mutations were detected in 6 (1.5%) of 388 cases; 5 cases with exon 9 mutations in E545K while one exon 20 mutation in H1047L. *PIK3CA* amplification is a frequent oncogenic alteration and associated with shorter survival, suggesting its role as a prognostic biomarker in resected ESCC. *PIK3CA* amplification may represent a promising therapeutic target for ESCC.

## INTRODUCTION

Esophageal cancer (EC) is the sixth most common cause of cancer death and the eighth most common cancer worldwide [1]. Despite the improvement in diagnosis and multidisciplinary treatment, the prognosis remains poor, even for patients who undergo complete resection [2]. Overall, though the conventional chemotherapy incorporating 5-fluorouracil, cisplatin, irinotecan and taxane, median overall survival remains 10-12 months with frequent toxicities [3]. The limited improvement with conventional therapies prompts us to explore the molecular biology and identify of prognostic and druggable biomarkers.

There are two main histologic types of esophageal cancer, esophageal adenocarcinoma (EAC) and esophageal squamous cell carcinoma (ESCC). EAC is associated with gastroesophageal reflux disease, Barrett's esophagus, and obesity [4–6], predominant in the United States and most other Western countries. However, ESCC dominates with 80% of all cases worldwide and predominant in Asia. In contrast to EAC, smoking and alcohol abuse contribute to the development of ESCC [7]. With those distinct biologic and epidemiologic differences, EAC and ESCC may need different therapeutic approaches. Over the past decade, molecular targeted therapy blocking important oncogenic pathway including human epidermal growth factor receptor-2 (HER2; also known as ERBB2) have led to remarkable progress in EAC including human epidermal growth factor receptor-2 (HER2; also known as ERBB2) [8]. Despite the improved outcome of EAC, ESCC still lacks therapeutically relevant predictive or prognostic target. A comparative genomic study revealed different genetic alterations between EAC and ESCC [9, 10]. Copy number gains of *SOX2, PIK3CA, CCND1*, and *FGFR1* were more frequent in ESCC than in EAC, implicating these genes as therapeutic targets for ESCC. Very recently, we reported that *FGFR1* amplification is frequently observed and an independent prognostic factor in resected ESCC [11]. Taken together, *PIK3CA* may also represent an attractive molecular target for ESCC.

Phosphoinisitide 3-kinase (PI3K)/Akt signaling pathway regulates cell proliferation, growth, survival, apoptosis, and glucose metabolism [12]. Activation of the PI3K pathway occurs upon engagement with mutation or amplification of *PIK3CA*, the p110α catalytic subunit of PI3K. Amplification and mutation of *PIK3CA* is generally associated with increased PIK3CA expression, PI3K activation, and phosphorylation of downstream Akt, supporting the oncogenic role of PI3K aberration. *PIK3CA* gene amplification was found in 10-30% of non-small cell lung cancer, breast cancer, colon cancer and head/neck cancer [13–16]. Activating somatic mutations (codons 542 and 545 in exon 9 and codon 1047 in exon 20) were also identified in various solid tumors [17]. Despite accumulating evidence of biologic role, only a few studies have reported the frequency of *PIK3CA* aberration in ESCC and its prognostic role is still controversial [18–22].

In this study, we evaluated the frequency of *PIK3CA* amplification and mutation in surgically resected ESCC. Furthermore, we also determined the prognostic impact of genetic aberration of *PIK3CA* in ESCC.

## RESULTS

### Patient characteristics

A total of 534 patients with curative esophagectomy were analyzed and their clinicopathologic features are presented in Table 1. The majority of patients were male (93.4%) with a median age of 65 years (range 31-90). Median tumor size was 4 cm and approximately half of the tumors were stage pT3 or pN0. Approximately two-thirds of cases (54.9%) were located in the lower esophagus and one-third in the middle esophagus. All patients received radical surgery, with evidence of pathologic stage I in 19.9%, stage II in 44.8%, and stage III in 35.4%. Two-thirds (63.9%) were moderately differentiated carcinoma, and more than half of patients located in the lower esophagus. The majority of patients were current (39.3%) or former (36.7%) smokers, and the median smoking dosage was 25 pack-years (range 0-150). Adjuvant treatment was given to 138 patients (25.8%), and 62 of these (44.9%) were treated with concurrent chemoradiotherapy. Adjuvant therapy was introduced in 4.7% for stage I, 21.3% for stage II, and 42.9% for stage III patients.

**Table 1:** Patient characteristics according to *PIK3CA* amplification

Characteristics	All patients		Amplification^*^				No amplification		P^†^
	No.	%	Ratio	Number	Total	(%)	No.	%	
No of patients	534	100	56	10.5			478	89.5	
Age, years									0.183
Median	65		66	65	66		65		(0.128)
Range	31-90		51-80	44-72	44-80		31-90		
Sex									0.851
Male	499	93.4	38	14	52	92.9	447	93.5	(0.979)
Female	35	6.6	3	1	4	7.1	31	6.5	
Tumor size, cm									0.148
Median	4.0		4.0	4.0	4.0		3.0		(0.093)
Range	0.2-14.5		1-9	1-8	1.0-9.0		0.2-14.5		
pT stage^‡^									0.026
T1	153	28.7	7	1	8	14.3	145	30.3	(0.020)
T2	113	21.2	7	7	14	25.0	99	20.7	
T3	252	47.2	27	7	34	60.7	218	45.6	
T4	16	3.0	0	0	0	0	16	3.3	
pN stage^‡^									0.426
N0	261	48.9	16	6	22	39.3	239	50.0	(0.540)
N1	244	45.7	21	9	30	53.6	214	44.8	
N2	18	3.4	3	0	3	5.4	15	3.1	
N3	11	2.1	1	0	1	1.8	10	2.1	
pTMN stage^‡^									0.053
I	106	19.9	7	1	8	14.3	98	20.5	(0.075)
II	239	44.8	12	8	20	35.7	219	45.8	
III	189	35.4	22	6	28	50.0	161	33.7	
Location									0.707
Cervical	13	2.4	2	0	2	3.6	11	2.3	(0.806)
Upper	70	13.1	7	2	9	16.1	61	12.8	
Middle	158	29.6	12	6	18	32.1	140	29.3	
Lower	293	54.9	20	7	27	48.2	266	55.6	
Histologic grade									0.158
Well	106	19.9	9	7	16	28.6	90	18.8	(0.080)
Moderate	341	63.9	28	6	34	60.7	307	64.2	
Poorly	87	16.3	4	2	6	10.7	81	16.9	
Smoking status^¶^									0.391
Never-smoker	128	24.0	11	5	16	28.6	112	23.4	(0.634)
Former smoker	196	36.7	13	3	16	28.6	180	37.7	
Current smoker	210	39.3	17	7	24	42.9	186	38.9	
Smoking dosage (pack-years)									
Median	25		30	15	26		25		0.924
Range	0-150		0-150	0-68	0-150		0-150		(0.645)
Adjuvant therapy									0.077
Yes	138	25.8	6	3	9	6.5	129	27.0	(0.099)
No	396	74.2	38	9	47	83.9	349	73.0	
*PIK3CA* FISH^§^									
Number (median, range)	2.2 (0-16.0)		4.6 (4.0-16.0)	5.6 (5.0-6.0)	5.4 (4.0-16.0)		2.1 (0-3.9)		<0.001
Ratio (mean, range)	1.1 (0-6.0)		2.5 (2.0-6.0)	1.5 (0.9-1.9)	2.3 (0.9-6.0)		1.1 (0-1.9)		<0.001

*Abbreviations*:

* PIK3CA amplification was defined as if one of the following criteria is fulfilled: (1) Ratio: *PIK3CA*/CEP3 ratio is ≥ 2.0, (2) Number: average number of *PIK3CA* signal per nucleus ≥ 5.0

† χ^2^ test, Fisher's exact test, or Mann-Whitney U test. Parenthesis indicates comparisons among ratio, numbers, and non-amplified groups

‡ Pathologic stage at the time of surgical resection was determined according to the American Joint Committee on Cancer (seventh edition) guidelines.

¶ Never-smokers; a lifetime smoking dose of fewer than 100 cigarettes; former smokers, those who have stopped smoking for more than 1 year; current smokers, those who currently smoke or have quit for less than 1 year.

§ *PIK3CA* numbers are average numbers of *PIK3CA* signals per nucleus, and ratios are *PIK3CA*/CEN3 ratios.

### Association of PIK3CA amplification status and clinicopathologic features

**Figure 1:**
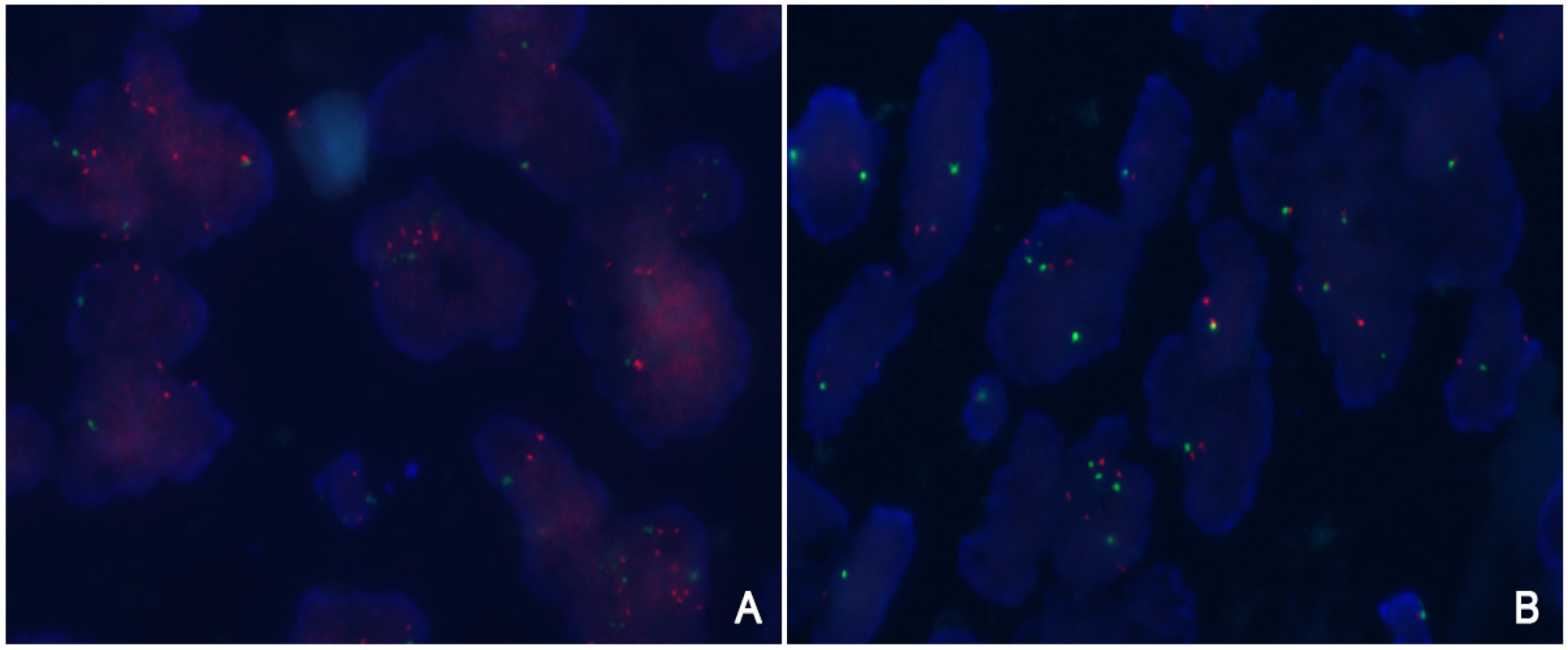
**Representative fluorescent in situ hybridization of tumors with A.** and without **B.** *PIK3CA* amplification. FISH analysis demonstrated an increase in the signals of *PIK3CA* (red signals) compared to reference CEN3 (green signals).

Among 534 patients, 41 (7.7%) of cases satisfied both criteria of *PIK3CA:* CEN3 ratio of ≥ 2.0 and an average number of *PIK3CA* signal per nucleus ≥5.0, whereas 15 (2.8%) cases only satisfied the criterion of *PIK3CA* ≥ 5.0 (Supplementary Figure S1). With our criteria, *PIK3CA* amplification was detected in 56 (10.5%) cases (Table 1, Figure 1). The median *PIK3CA* gene copy number was 5.4 (range, 4.0-16.0) and 2.1 (range, 0-3.9) in *PIK3CA* amplified and non-amplified groups, respectively. The mean *PIK3CA*/CEN3 ratio was 2.3 (0.9-6.0) for the amplification group and 1.08 (0-1.9) for the no amplification group.

There was no significant difference in *PIK3CA* amplification according to age, sex, tumor size, location, histologic grade, smoking and adjuvant therapy as shown in Table 1. However, *PIK3CA* amplification was significantly associated with a higher T-stage (P=0.026). Although only marginal statistical significance was achieved, there was a positive association between *PIK3CA* amplification and higher pathologic stage (P=0.053). Based on the pattern of amplification (*PIK3CA:* CEN3 ratio of ≥ 2.0 and signal per nucleus ≥5.0 *vs. PIK3CA* signal per nucleus ≥5.0), there was similar statistical significance compared to no amplification group.

### Prognosis according to *PIK3CA* amplification

The 5 year DFS and OS rates for all patients were 46.3% and 59.8% with a median follow-up time of 56.4 months. The 5-year DFS rate according to pTNM stages was 64.6% in stage I, 55.4% in stage II, and 28.9% in stage III. The 5-year OS rate according to pTNM stages were 76.7% for stage I, 61.3% for stage II, and 32.4% for stage III.

**Figure 2:**
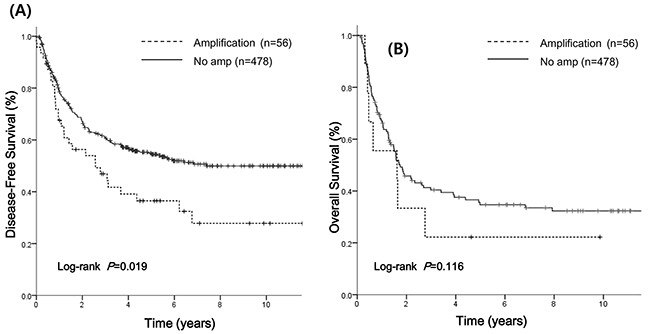
Survival analysis according to *PIK3CA* amplification **A.** Median disease-free survival (DFS) was 33.4 months in the *PIK3CA* amplification group and 63.1 months in the no amplification group. **B.** Median overall survival (OS) was 52.1 months in the amplification group and 96.5 months in the no amplification group.

*PIK3CA* amplification showed a significantly shorter DFS compared with that of non-amplified group (33.4 *vs* 63.1 months, P=0.019, Figure 2A). Though the statistical insignificance probably due to small sample size, *PIK3CA* amplification showed a tendency of shorter OS than no amplification (52.1 *vs* 96.5 months, P=0.116, Figure 2B). In the Cox proportional hazard model adjusted for gender, tumor location, pathologic stage, histologic grade and adjuvant treatment, *PIK3CA* amplification was significantly associated with a shorter DFS (adjusted hazard ration [AHR] 1.53; 95% CI, 1.10-2.17; P=0.02, Table 2). There was trend toward worse DFS for the pattern of amplification (AHR 1.53; 95% CI 1.1-2.3 for *PIK3CA:* CEN3 ratio of ≥ 2.0 *vs* AHR 1.32; 95% CI, 0.97-2.56; *PIK3CA* signal per nucleus ≥5.0; P=0.05) compared to non-amplified group. There was no significant difference in OS according to *PIK3CA* amplification, gender and adjuvant treatment in multivariate analysis. Regarding the prognostic role of *PIK3CA* amplification according to pathologic stage, there were no significant difference with *PIK3CA* amplification in DFS (5-year DFS 38.1% *vs* 57.3% in stage II [P=0.074]; 16.1% *vs* 28.8% in stage III [P=0.356], Supplementary Figure S2).

**Table 2:** Survival outcome in multivariate analysis

Variable	Category	DFS				OS		
		Univariate P value	HR	95% CI	P	HR	95% CI	P
**Sex**	Female *vs* Male (ref)	0.32	0.80	0.48-1.33	0.39	0.82	0.46-1.41	0.47
**Location**	Lower *vs* upper/middle	0.07	1.63	1.13-2.35	0.008	1.53	1.04-2.24	0.03
Pathologic stage^*^	II/III *vs* I (ref)	<0.001	2.32	1.80-2.99	<0.001	2.79	2.14-3.66	<0.001
**Histology**	Poor *vs* well/moderate (ref)	0.06	1.34	0.99-1.80	0.06	1.32	0.96-1.80	0.08
**Adjuvant treatment**	Yes *vs* no (ref)	0.05	1.22	0.93-1.61	0.16	1.05	0.79-1.39	0.75
***PIK3CA* amplification**	Amplification *vs* no amplification (ref)	0.01	1.53	1.10-2.17	0.02	1.21	0.83-1.77	0.30

*Abbreviations*: DFS, disease-free survival; OS, overall survival; ref, reference; amp, amplification;

* Clinical stage at the time of initial diagnosis was determined according to the American Joint Committee on Cancer (seventh edition) guidelines

We next explored the role of adjuvant treatment according to *PIK3CA* amplification. We additionally analyzed survival outcome for two subgroups: one group without treatment (n=396) and another group with adjuvant chemotherapy and/or radiotherapy (n=138). In the adjuvant group, *PIK3CA* amplification showed an inferior DFS but it was statistically insignificant (median DFS 21.0 *vs* 22.8 months, P=0.39, Supplementary Figure 3A). Among the patients without adjuvant treatment, the *PIK3CA* amplification group had a significantly shorter DFS compared with non-amplified group (median DFS 33.5 vs 96.5 months, P=0.011, Supplementary Figure S3B).

### *PIK3CA* mutations

**Figure 3:**
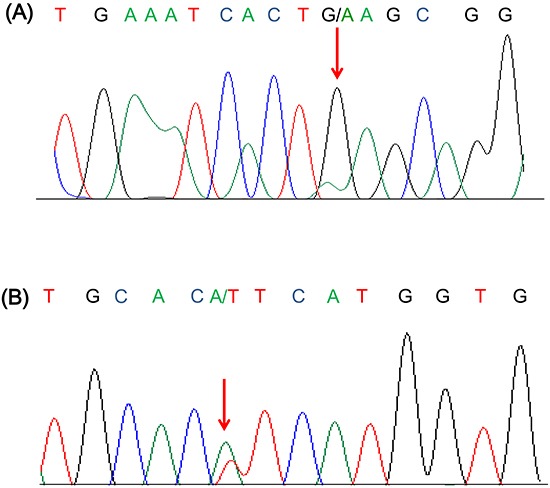
Representative sequence chromatogram of **A.** E545K (G1634A) and **B.** H1047L (A3140T) mutation.

Among the 526 patients, we examined *PIK3CA* exon 9 and 20 mutations using direct sequencing in 388 cases with FFPE tissues available. *PIK3CA* mutations were detected in 6 (1.5%) of 388 cases; 5 cases in exon 9 only, and 1 case in exon 20 only. The clinicopathologic characteristics of the 6 patients with *PIK3CA* mutations are listed in Table 3. *PIK3CA* exon 9 mutations were identified in 5 tumors of E545K (GAG to AAG), while exon 20 mutation was identified in H1047L (CAT to CTT) (Figure 3). No co-occurrence of exon 9 and exon 20 mutations was identified. Of 6 *PIK3CA* mutants, the single case with exon 20 mutation satisfied the criteria of *PIK3CA* amplification with *PIK3CA*/CEN3 ratio of 2.5. The patient was diagnosed as stage IIB, upper ESCC with well-differentiated histology. He had experienced recurrence within 6 months after esophagectomy and died of disease. Though the statistical insignificance, all 6 cases were high T stage (5 with T3 and 1 with T4) and 4 out of 6 cases (66.7%) were lymph node negative. Furthermore, all tumors with *PIK3CA* mutations originated from the mid-to-lower esophageal region. No prognostic difference in DFS (*P*=0.876) and OS (*P*=0.695) was detected according to the presence of *PIK3CA* mutation.

**Table 3:** Clinicopathologic characteristics of 6 patients with *PIK3CA* mutation

No.	Mutation	Domain	Age/Sex	Stage	Histology	Recur	Death	*PIK3CA* amplification
1	E545K	Helical	Male/56	IIA (T3N0)	Well	No	No	No
2	E545K	Helical	Male/54	IIB (T3N0)	Moderate	Yes	Yes	No
3	E545K	Helical	Male/65	IIIA (T3N1)	Well	No	No	No
4	E545K	Helical	Male/61	IIIA (T3N1)	Moderate	Yes	Yes	No
5	E545K	Helical	Male/59	IIIA (T4N0)	Moderate	No	No	No
6	H1047L	Kinase	Male/66	IIB (T3N0)	Well	Yes	Yes	Amplified

## DISCUSSION

We conducted this study to examine the frequency and prognostic impact of *PIK3CA* amplification among curatively resected ESCCs. To our knowledge, this is the first study of *PIK3CA* amplification in a large cohort of East Asian ESCC patients. We found that PIK3CA amplification is a common genetic event and an independent poor prognostic factor in ESCC.

Though the known therapeutic options in EAC such as HER2 inhibitor, ESCC still lacks therapeutically relevant genetic alterations. In the era of personalized medicine, EAC and ESCC show different genetic aberrations for therapeutic strategies. A recent comparative genome study reported focal regions of DNA amplification or loss including *SOX2, PIK3CA, CCND1, and FGFR1* are more frequent in ESCC than EAC [9]. A study of DNA copy number profiles also revealed frequent alterations of the 3q22-26 regions containing *PIK3CA* in ESCC patients [10]. Therefore, PIK3CA may be a putative drive gene for ESCC with the available therapeutic agents and examining *PIK3CA* aberrations in a large cohort of ESCC is worthwhile.

Activation of the PI3K pathway, generally as a result of *PIK3CA* amplification, has been reported in upper aerodigestive tract cancers including 12% of lung squamous cell carcinoma (SCC) [23], 18.2% of nasopharyngeal carcinoma [24], 20% of oropharyngeal SCC [25], and 32.2% of HNSCC [16]. For esophageal carcinoma, one study demonstrated 26.7% of *PIK3CA* gene amplification in ESCC [18]. With FISH analysis, we found an overall 10.5% frequency of *PIK3CA* amplification.

Regarding the prognostic significance of *PIK3CA* amplification, previous reports yielded controversial results. In nasopharyngeal carcinoma, *PIK3CA* amplification was strongly associated with distant metastasis, lymph node involvement, advanced tumor stage, and ultimately with reduced overall survival [24]. For non-lymph node metastatic HNSCC, patients with *PIK3CA* amplification showed earlier recurrence than those without (10% *vs* 31% disease free at 2 years) [16]. Angulo *et al* reported that *PIK3CA* amplification was significantly more frequent in lung SCC compared with adenocarcinoma (42% *vs* 3%, P<0.001), however, no association was found with other clinicopathologic characteristics [15]. Clinicopathologic heterogeneity including primary tumor, pathologic stage, and adjuvant treatment, may contribute to the controversial results. Furthermore, conclusions based on small sample sizes may lead to inconsistent results. In our study, by carefully assessing a large cohort of ESCC cases, we were able to clarify the prognostic value of *PIK3CA* amplification in a homogenous ESCC patients. Therefore, *PIK3CA* amplification was significantly associated with shorter DFS regardless of sex, histologic grade, and adjuvant therapy, implying a potential role as an independent negative prognostic factor in curatively resected ESCC.

To investigate *PIK3CA* gene copy number gain, most commonly used methods are real-time quantitative polymerase chain reaction (PCR) and *in situ* hybridization [15, 16, 18, 24, 26, 27]. With PCR studies, it is difficult to compare results among studies because of different cut-off value; some studies used copy number more than 4 [16, 28] whereas other group considered 2 or 3 copies as amplification [18, 23]. In contrast, relatively consistent criteria are used for FISH studies. The majority of studies defined *PIK3CA* amplification as *PIK3CA*/CEP3 ≥ 2 [25–27] and another group defined it as copy number more than 5 [15]. In our large cohort study, we used a combination of these criteria and demonstrated a significant difference in prognostic value. In our study, 26.7% of cases with copy number ≥5 might not have been considered amplified if only the ratio criterion of ratio ≥ 2.0 was used. FISH is also more powerful than PCR by allowing visualization of individual cancer cells in a routine clinical environment. Identification *PIK3CA* amplification by FISH analysis may contribute to easy subgroups selection. Our criteria should be further validated in other solid tumors and in clinical trials with PI3K inhibitors.

Hotspot mutations of *PIK3CA* in exon 9 and exon 20 have been shown to increase lipid kinase activity, leading to downstream activation [29]. The frequency of *PIK3CA* mutation has been variously reported as 2.2 to 21% of ESCC cases, with controversial prognostic value [19–22]. Mori *et al* identified a frequency of 2.2% using PCR-based direct sequencing [30] and Hou *et al* reported a frequency of 12.5 % with mutant-enriched liquid chip technology [19]. More recently, Shingaki *et al* identified *PIK3CA* mutations in 21% of cases by employing a pyrosequencing approach [22]. Here, we report *PIK3CA* mutations in 1.6% of ESCCs using standard, bidirectional Sanger sequencing. Different sequencing methodologies may have an important influence on the reported frequencies. The limited sensitivity of Sanger sequencing may result in an apparent the low frequency and further studies to compare these multiple methods are warranted.

Elevated PI3K signaling correlates with *PIK3CA* mutation and/or amplification. In addition it is associated with increased activity of PI3K effector protein kinase B (PKB) [26] and pAkt [31], suggesting that these may be susceptible to PI3K inhibitors. In lung cancer cell lines, 37% of squamous cell harbored *PIK3CA* amplification and they were sensitive to PI3K inhibitor GDC-0941 with less than 1μmol/L of IC50 [31]. In a preclinical platform from Cancer Cell Line Encyclopedia, *PIK3CA*-amplified tumors were sensitive to BYL719, a PI3K α-selective inhibitor [32]. Cell lines with *PIK3CA* amplification was positively associated with BYL719 sensitivity (P=0.0037) and tumor-bearing mice with *PIK3CA* amplification responded to BYL719, leading to a response rate of −18% (lung cancer) and −80% (gastric cancer). Despite the preliminary data, recent phase I trials have explored the potential predictive role of the *PIK3CA* gene. Pan-PI3K (BKM120 and GDC-0941) and α-selective inhibitors (BYL719 and GDC-0032) demonstrated responses and prolonged stable disease in patients with *PIK3CA* mutation [33–36]. In addition to *PIK3CA* mutation, *PIK3CA* amplification was positively associated with sensitivity to BYL719 in *PIK3CA* wild type cell lines [32]. In a phase I trial of GDC-0941, a heavily treated ovarian cancer patient with *PIK3CA* amplification achieved disease stabilization for 4 months with significant pharmacodynamic changes [37]. To date, clinical trials with PI3K inhibitors have been reported in un-selected patients, and current trials with PI3K inhibitors are for patients with *PIK3CA* gene alterations are ongoing (ClinicalTrials.gov number NCT01928459 and NCT01608022). In addition, as shown in our study, frequency of *PIK3CA* amplification increases from 14.3% (stage I) to 50% (stage III) indicating more advanced patients may have benefit from PI3K inhibitors. Our results require further validation with PI3K inhibitors in clinical trials.

The main limitations of our study include its retrospective nature and patient selection. Therefore, our findings should be validated in an independent cohort and response data to PIK3CA-targeted therapies in the future clinical trials.

In conclusion, in our large cohort study, we demonstrated that *PIK3CA* amplification is an independent poor prognostic factor in resected ESCC. Our findings also provide strong implication that *PIK3CA* amplification and mutation is a promising therapeutic target in ESCC.

## MATERIALS AND METHODS

### Patients and tissue samples

A total of 534 patients with ESCC who underwent radical esophagectomy at Severance Hospital and Samsung Medical Center, Seoul, Korea between 2002 and 2010 were enrolled in this study. The criteria used for patient selection included (1) surgically resected SCC of the thoracic esophagus (R0 resection), (2) availability of primary tumor tissue, (3) no distant metastasis, and (4) no preoperative treatment. Tumor samples were available for 664 patients, of which we excluded 107 cases (16.1%) who received neoadjuvant treatment. Twenty-three patients (3.5%) were excluded because of incomplete survival follow-up. All diagnosis were reviewed by two experienced pathologists (Y.L.C. and H.K.) and confirmed by hematoxylin and eosin staining. Paraffin-embedded tumor specimens were used to construct a tissue microarray with three representative cores of 2-mm-diameter.

Clinicopathologic characteristics and survival outcome was collected by reviewing the medical records. Staging was determined using the 7th edition of the American Joint Committee on Cancer guideline of tumor, node, and metastasis (TNM) classification. Smoking status of never-smoker, former smoker, and current smoker were defined as in previous studies (23). The study was approved by the institutional review board of Severance Hospital and Samsung Medical Center.

### *PIK3CA* fluorescence *in situ* hybridization

To assess the presence of *PIK3CA* amplification, we performed fluorescence in situ hybridization (FISH) on tissue microarrays. PIK3CA (Spectrum Green) and CEP3 (Spectrum Orange) FISH was performed as per manufacturer's recommendation. (Abbott Molecular, Abbott Park, IL, USA). Evaluation was performed independently by two experienced pathologists (Y.L.C. and H.K.K) blinded to clinical information, and at least 100 nuclei per case were evaluated. Based on the previous studies(24-27), *PIK3CA* amplification was defined as fulfillment of one of the following criteria: (1) PIK3CA/CEP3 ratio ≥ 2.0 and (2) average number of PIK3CA signals per nucleus ≥ 5.0

### Mutation analysis

Genomic DNA was extracted from 388 formalin-fixed paraffin-embedded (FFPE) tissue specimens using a QIAamp DNA Micro kit (Qiagen, Valencia, CA, USA) according to the manufacturer's instruction. The extracted DNA was used in a PCR amplification reaction with primers were designed to amplify the following regions at codon E542, E545 and H1047; exon 9; 5′;- AGAGACAATGAATTAAGGGAAAATGAC-3′;; 5′;- TTTAGCACTTACCTGTGACTCCA-3′;, exon 20; 5′;-TATTCGACAGCATGCCAATC-3′;; 5′;- TGTGTGGAAGATCCAATCCA-3′;,.

PCR was carried out with the following conditions: initial denaturation at 95°C for 10 min, followed by 45 cycles of 95°C for 30 s, 54°C for 60 s, 72°C for 45 s and a final polymerization step of 72°C for 5 min in a GeneAmp PCR system 2720 (Life technologies, CA, USA). The amplified DNA product was visualized by gel electrophoresis and PCR products were sequenced using the Big Dye terminator sequencing kit (Life technologies, CA, USA) according to the manufactures’ instruction. Sequence reactions were the subjected to electrophoresis on an Applied Biosystems 3130XL DNA Analyzer (Life Technologies, CA, USA).

### Statistical analysis

We analyzed the association between *PIK3CA* amplification status and clinical significance using the χ2 test or Fisher's exact test. We also assessed the prognostic value of PIK3CA amplification on survival outcome using Kaplan-Meier curves with a log-rank test. Disease free survival (DFS) was defined from the time of surgery to initial relapse or death. Overall survival (OS) was measured from the time of surgery to death or the last follow-up date, and 95% confidence intervals (CIs) were evaluated by survival analysis using the Kaplan-Meier method. Statistical significance was defined as P < 0.05 for all analyses. Multivariate analysis was done using Cox regression analysis for following variables: gender, location, pathologic stage, histology, adjuvant treatment, and PIK3CA amplification status. All statistical analysis was performed using SPSS version 18.0 (SPSS, Chicago, IL, USA).

## SUPPLEMENTARY FIGURES


