# Supplementary material for: PIK3CA amplification is associated with poor prognosis among patients with curatively resected esophageal squamous cell carcinoma
Source: Oncotarget. 2016 Apr 15;7(21):30691–701. doi: 10.18632/oncotarget.8749 (PMC5058710; doi:10.18632/oncotarget.8749)
Supplement: Supplementary file 1 [file oncotarget-07-30691-s001.pdf]

PIK3CA amplification is associated with poor prognosis among patients with curatively resected esophageal squamous cell carcinoma

SUPPLEMENTARY FIGURES

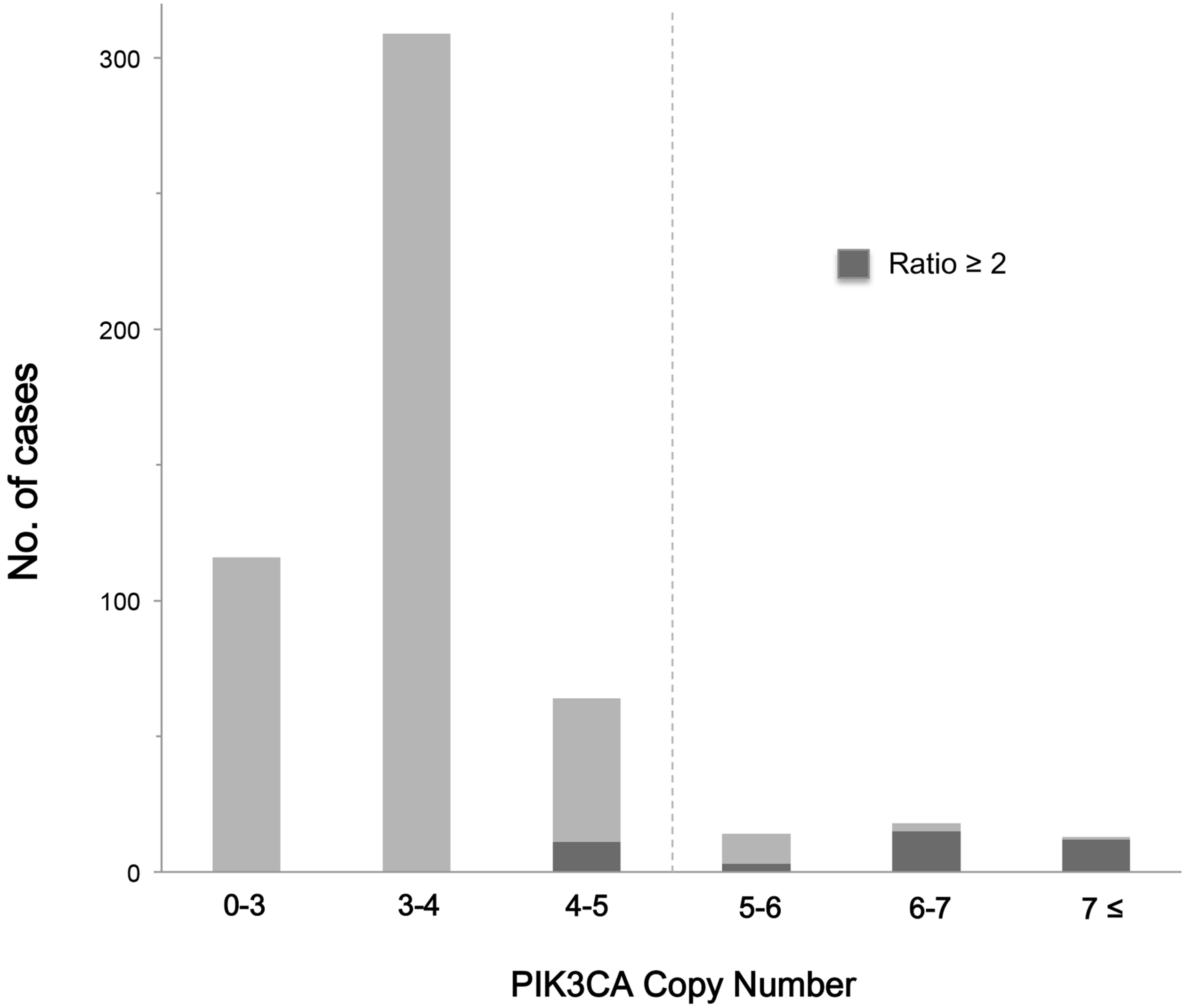

Supplementary Figure S1: Detailed number of cases according to PIK3CA amplification status.

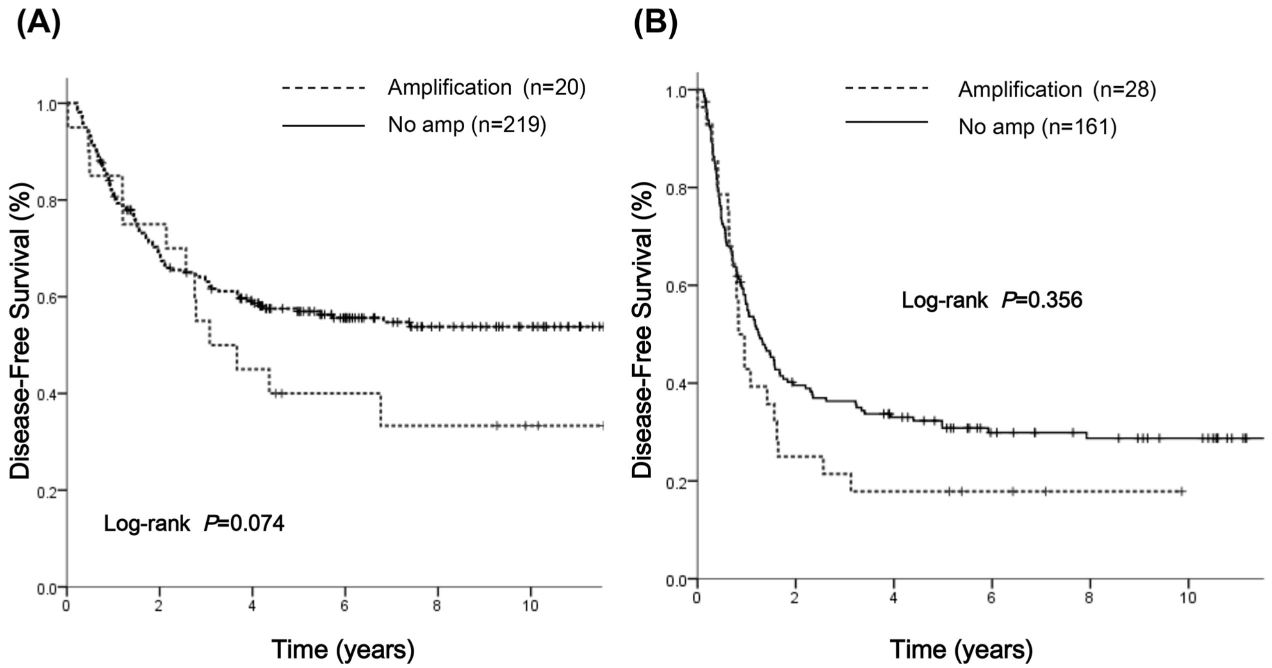

Supplementary Figure S2: Prognostic role of *PIK3CA* amplification according to pathologic stage II A. and III **B.**

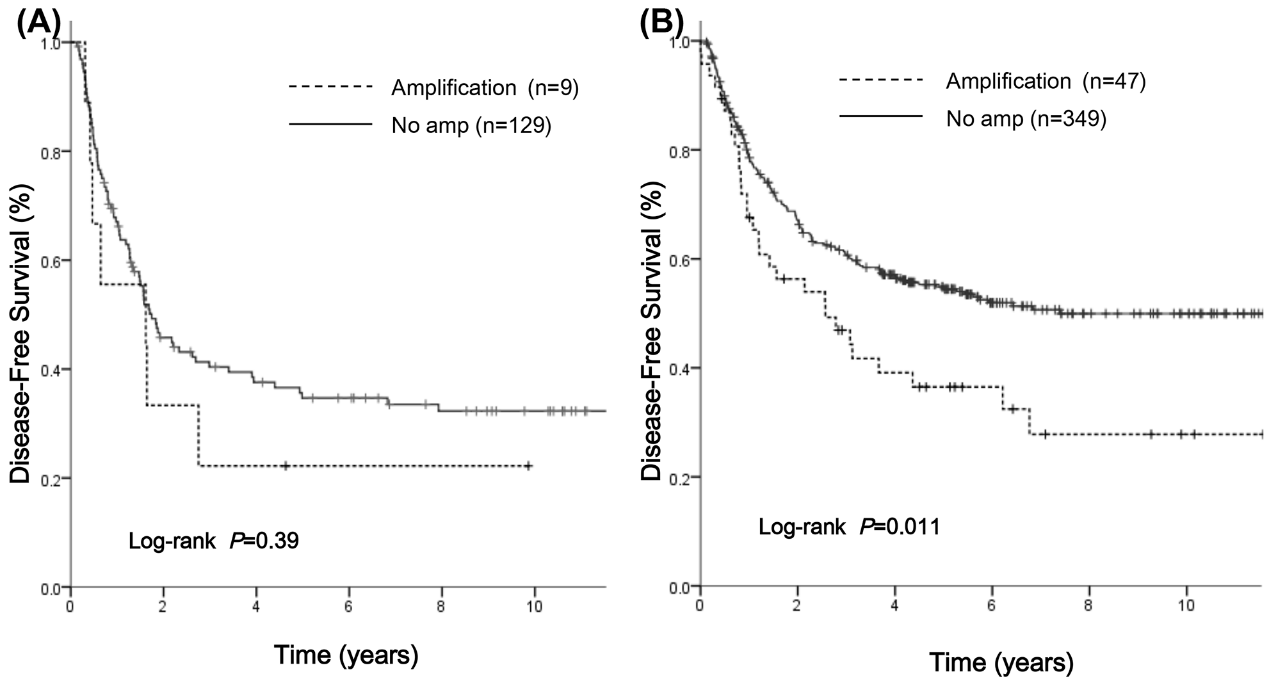

Supplementary Figure S3: Prognostic role of *PIK3CA* amplification with A. or without **B.** adjuvant chemotherapy.
